# The height-, weight-, and BMI-for-age of Polish school-aged children and adolescents relative to international and local growth references

**DOI:** 10.1186/1471-2458-10-109

**Published:** 2010-03-04

**Authors:** Zbigniew Kulaga, Mieczysław Litwin, Marcin Tkaczyk, Agnieszka Różdżyńska, Katarzyna Barwicka, Aneta Grajda, Anna Świąder, Beata Gurzkowska, Ewelina Napieralska, Huiqi Pan

**Affiliations:** 1 Public Health Division, The Children's Memorial Health Institute, Warsaw, Poland; 2 Department of Research, The Children's Memorial Health Institute, Warsaw, Poland; 3 Department of Nephrology and Dialysis, Polish Mother's Memorial Hospital Research Institute, Łódź, Poland; 4 Anthropology Division, Department of Paediatrics, The Children's Memorial Health Institute, Warsaw, Poland; 5 MRC Centre of Epidemiology for Child Health, UCL Institute of Child Health, UK

## Abstract

**Background:**

The growth of children is an indicator of health and society's wellbeing. Growth references are useful in monitoring a child's growth, which is a very important part of child care. Poland's growth references are not updated regularly. Although several growth reference ranges have been developed in Poland over recent years, sampling was restricted to urban populations of major cities. The aim of this study was to assess how well Polish children match with, or diverge from, regional charts and to compare them with international growth references.

**Methods:**

Four Polish and two international (WHO 2007 and USCDC2000) growth references were used to calculate the height, weight and BMI z-scores in a recent, large, population-representative sample of school-aged children and adolescents in Poland. The distributions of z-scores were analysed with descriptive and inferential statistical methods.

**Results:**

Mean height z-scores calculated with the use of the WHO 2007 and USCDC2000 references were positive and significantly different from zero over the entire age range. The mean height z-score was closest to zero in the Poznan reference for boys (0.05) and Warszawa reference for girls (0.01). Median weight z-scores were positive under all weight references over the entire age range with only the exception of 18-year-old girls' weight z-score calculated relative to USCDC2000. Median BMI z-scores were positive in males in early childhood, decreasing with age. In the case of girls, the median BMI z-score calculated using WHO 2007 and USCDC2000 was close to zero in early childhood, decreased in adolescents and reached minimum values at age 18 years. Median BMI z-scores calculated with the use of the Lodz reference fluctuated between 0.05 and 0.2 over the studied age range.

**Conclusions:**

In this contemporary sample of Polish school-aged children, distributions of height, weight and BMI differed from those of children from the international growth references. These differences should be considered when using the references. There exist certain limitations to the analysis of height, weight, and BMI z-scores when Polish regional references are used.

## Background

A child's growth is an indicator of health [1,2] and society's wellbeing [3,4]. Monitoring growth to identify health- or nutrition-related problems is an important task of health care providers. In practice, growth references are used: 1) by doctors and nurses involved in the care of individual children as a part of medical assessment to evaluate the growth status of a child, and 2) as a public health tool, to summarize and compare anthropometry among groups of children [5].

Poland does not have its own growth reference data that have been constructed with a representative national sample of children and adolescents and regularly updated, for example, in ten-year intervals. Although several growth references have been developed in Poland in recent years, sampling was restricted to the urban populations of major cities and, in the case of some of the references, sampling was not random. Due to the limited representativeness of Polish local growth charts, the recently updated World Health Organization (WHO) international height, weight, and BMI references for children and adolescents aged 5-19 years (WHO 2007) [6] are of interest as potentially useful for the Polish population, as are the reference values from the Centers for Disease Control and Prevention (CDC) [7] (USCDC2000). The aim of the present analysis was to assess how well Polish children match with, or diverge from, regional and international growth charts. We have addressed these questions using data from a representative sample of Polish schoolchildren (7 to 18 years of age).

## Methods

The analyzed data were collected in the course of the OLAF study (PL0080) in which the reference blood pressure ranges were elaborated for Polish children and adolescents. The present analysis was limited to a cross-sectional sample of school children who were examined between November 2007 and March 2009. Study participants (children and adolescents 6.5-18.5 years of age) were randomly selected using two-stage sampling. Primary units (schools) were sampled from an all-schools-in-Poland sampling frame; sampling was stratified by urban/rural area. In the second stage, all pupils in the required age ranges within the sampled schools comprised the sampling frame. Pupils in schools were selected for the survey by stratified random sampling, the stratification variables being classes. All subjects and their parents (in the case of subjects under 18 years of age) gave their informed consent to participate in the study (subjects over 16 years of age and parents gave written consent). Approval of The Children's Memorial Health Institute Ethics Committee to conduct the study was obtained before the study commenced.

The medical history of the study participants, including past and present diseases, as well as medications used, was taken from the parents. The general health status of each subject was assessed by a physician. Height was measured in duplicate (in case of a difference between measurements exceeding 4 mm, a third measurement was taken) using a stadiometer (SECA 214) in the standing position (with no shoes), to the nearest millimeter. Body weight was recorded in light underwear to the nearest 0.05 kg, using a digital, medical scale (Radwag WPT 100/200). Body mass index was calculated as body weight divided by height in meters squared. The exact ages of the participants were calculated from birth and examination dates. There were 13 015 children and adolescents enrolled in the study (response rate 0.70). In our analysis we excluded height measurements from the OLAF study if the child was recorded as having a posture deficiency, genetic syndrome, cancer, or other chronic disease (230 cases). The weight measurements were missing in the OLAF study sample for 1 boy and 3 girls. Thus, 6227 boys and 6558 girls aged 6.5 to 18.5 years from the OLAF study were included in the analysis of height and 6226 boys and 6555 girls in the analysis of weight and BMI. One third (33%) of all of the children and adolescents in the sample lived in rural areas.

Four Polish growth reference ranges were compared with those of the WHO 2007 [6] and USCDC2000 [7] growth charts. The four Polish growth charts included in the present analysis were developed for urban populations of: Warszawa [8], Poznan [9], Krakow [10], and Lodz [11]. The Polish growth charts are referred to herein by the name of the city for which they were developed. The growth references for Krakow and Lodz were constructed with the LMS method [12], which is equivalent to the BCPE method used in the construction of the WHO 2007 references, while the USCDC2000 used a process similar to the LMS method.

All six growth references under consideration were used to calculate height z-scores. Only three weight and BMI references: WHO 2007, USCDC2000, and Lodz, were used to calculate z-scores for the OLAF sample, as the Warszawa and Poznan weight and BMI references did not account for skewness of weight and BMI, while Krakow did not provide the L, M, S parameters for the calculation. Z-scores relative to the USCDC2000 were calculated with the SAS code downloaded from the CDC web site http://www.cdc.gov/nccdphp/dnpa/growthcharts/resources/sas.htm. Z-scores relative to the WHO 2007 were calculated with the SAS code provided by the WHO Anthro Team. The z-score (z) for a given height, weight, and BMI measurement (X) relative to the Lodz reference was calculated as: z = ((X/M)^L-1)/L/S if L ≠ 0 or z = log(X/M)/S if L = 0 [12]. Height z-scores relative to the Krakow, Warszawa and Poznan references were calculated according to the formula: z = (X-mean)/SD.

A child was considered stunted (low height-for-age) if the height-for-age z-score was below -2. The means and standard deviations of z-scores of height and percentage of stunted children were calculated separately for each sex and for each height-for-age chart. The normality of distributions of estimated height z-scores were assessed with the Kolmogorov-Smirnov test. Differences from zero of the means of height z-scores were analyzed with Student's t-test for the whole age range (7 to 18 years of age) and separately for each year of age. Differences of height z-score means between reference ranges were analyzed with the paired t-test. Differences in the distribution of stunting according to sex and reference range were tested with the McNemar test. Differences in the prevalence of stunting between genders were tested with the chi square test. Due to the skewed nature of weight and BMI distributions, medians and inter-quartile ranges (IQRs) are presented by sex and age. Data were processed with the MsAccess database and MsExcel spreadsheet. All analyses were conducted with SAS 9.1 for Windows.

## Results

Additional file 1: Table S1 shows the height, weight, and BMI summary statistics of the OLAF study subjects included in the analysis. Due to the skewed nature of weight and BMI distributions, medians and inter-quartile ranges (IQRs) are presented.

Table 1 shows the means of height z-scores, which, calculated using the WHO 2007 and USCDC2000 height references, were positive and higher than the means calculated using Polish regional references. The confidence interval of mean height z-scores included zero only for the Warszawa growth chart for girls. For all other studied reference ranges, statistically significant differences from zero of the mean height z-scores (p = 0.0001) were demonstrated for both sexes, however, the Poznan and Warszawa mean height z-scores were close to zero (from -0.06 to 0.08).

**Table 1:** Polish children and adolescents (the OLAF study sample) mean height z-scores relative to the six references and statistical testing

reference	N	Mean	95% CI	SD	p	**Min**.	**Max**.
boys							
**WHO 2007**	6227	0.50	0.47 0.52	0.99	<.0001	-3.03	4.37
**USCDC2000**	6227	0.45	0.43 0.48	0.97	<.0001	-2.79	4.02
**Krakow**	6227	0.17	0.15 0.20	1.02	<.0001	-3.37	3.85
**Warszawa**	6227	0.08	0.05 0.11	1.06	<.0001	-3.63	4.07
**Poznan**	6227	0.05	0.03 0.08	1.09	0.0001	-3.73	4.08
**Lodz**	6227	0.14	0.11 0.16	1.04	<.0001	-3.66	3.88
**girls**							

**WHO 2007**	6558	0.34	0.32 0.37	0.95	<.0001	-3.47	4.29
**USCDC2000**	6558	0.33	0.31 0.36	0.94	<.0001	-3.03	3.95
**Krakow**	6558	0.09	0.07 0.12	1.03	<.0001	-3.34	4.33
**Warszawa**	6558	0.01	-0.02 0.03	1.05	0.6338	-3.73	4.19
**Poznan**	6558	-0.06	-0.09-0.03	1.11	<.0001	-3.86	4.57
**Lodz**	6558	0.15	0.13 0.18	1.03	<.0001	-3.49	4.43

**Figure 1:**
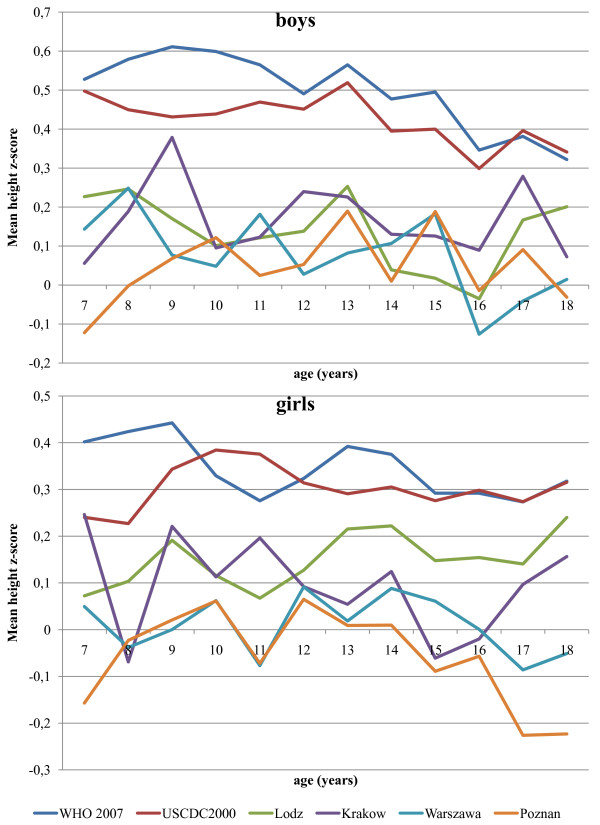
**Polish children (the OLAF study sample) means of height z-scores relative to six reference growth charts**.

The mean height z-score difference from zero was systematically positive over the whole age range (both boys and girls) (Figure 1) and statistically significant in relation to the WHO 2007 and USCDC2000 height references in each year of age (Additional file 2: Table S2).

With the exception of z-scores calculated using the Lodz height reference, mean height z-scores were higher in males compared with females for all other tested growth charts. The difference between genders was statistically significant (p < 0.01) for the WHO 2007, USCDC2000, Warszawa, Krakow, and Poznan growth charts.

The differences between mean z-scores for height were statistically significant (p < 0.0001) compared with the WHO 2007 and USCDC2000 charts for each of the Polish growth references and between each pair of Polish reference growth charts.

**Figure 2:**
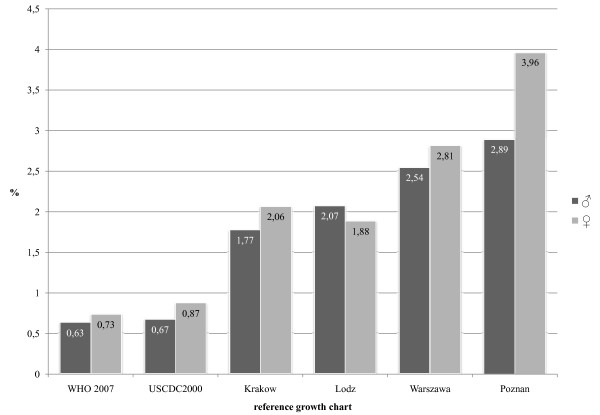
**Prevalence of stunting among Polish school children (the OLAF study sample) using the six references**. Stunting was defined as height z-scores <-2.

The use of WHO 2007 and USCDC2000 height-for-age references resulted in an estimated stunting (height-for-age z-score less than -2) prevalence of below 1% among OLAF study participants. Figure 2 presents stunting rates in the OLAF study sample calculated using the six references. In general, the rate of stunting was higher in girls than in boys, with the exception of the Lodz height reference. The six references showed differences in stunting rates: the lowest were in the WHO 2007 and USCDC2000 references, and the highest in Poznan. There were significant differences (p < 0.001, McNemar tests) in the prevalence of stunting when Polish references were compared with the WHO 2007 and USCDC2000 growth charts.

The distributions of weight and BMI z-scores calculated relative to the WHO 2007 and Lodz growth references were right-skewed, whereas the distributions of the OLAF study sample weight and BMI z-scores calculated relative to the USCDC2000 growth references were close to normal with a negative skew tendency. The WHO 2007 does not provide weight reference values for children older than 10 years, thus z-scores were calculated for ages from 7 to 10 years only. Table 2 shows descriptive statistics of weight and BMI z-score by sex calculated with the use of two international and one Polish growth reference.

**Table 2:** Polish children and adolescents (the OLAF study sample) median weight and BMI z-scores relative to the WHO 2007, USCDC2000, and Lodz growth references

reference	weight z-score					BMI z-score				
		
	N	**Min**.	Median	**Max**.	IQR	N	**Min**.	Median	**Max**.	IQR
boys										
**WHO 2007**	1837	-3.08	0.52	5.92	1.72	6226	-4.18	0.11	5.91	1.65
**USCDC2000**	6226	3.71	0.30	3.67	1.38	6226	-5.03	0.06	2.96	1.51
**Lodz**	6226	-3.04	0.10	3.71	1.31	6226	-3.93	0.03	3.64	1.34
**girls**										

**WHO 2007**	1778	-3.17	0.29	5.06	1.57	6555	-3.90	-0.07	4.54	1.41
**USCDC2000**	6555	-4.36	0.10	3.27	1.26	6555	-4.39	-0.06	2.73	1.32
**Lodz**	6555	-4.40	0.18	3.94	1.33	6555	-4.24	0.14	3.62	1.30

**Figure 3:**
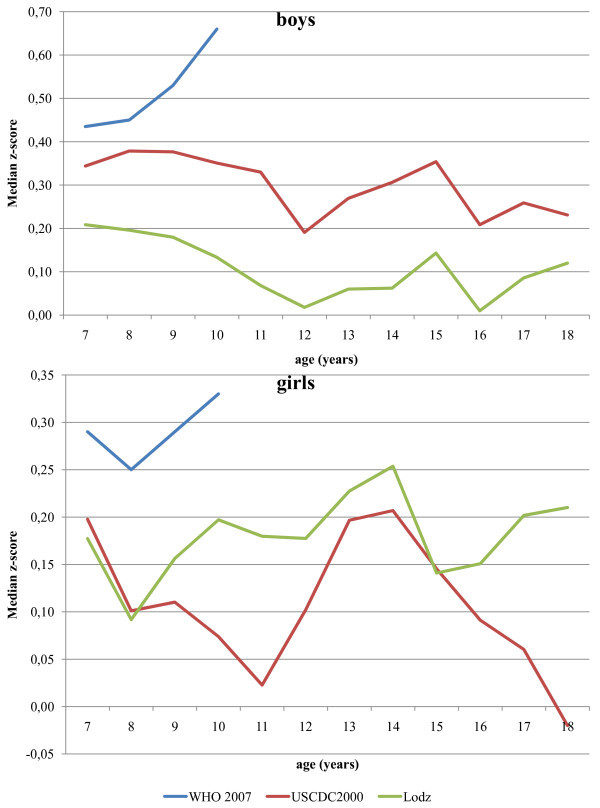
**Polish children (the OLAF study sample) median weight z-scores calculated using three weight-for-age references**.

Under all three weight references, over the entire age range, median weight z-scores were positive, with only the exception of 18-year-old girls' weight z-score relative to the USCDC2000. In both sexes, the median weight z-score calculated using the WHO 2007 reference was higher compared with the USCDC2000 and Lodz weight references and, in contrast with the USCDC2000 and Lodz, the median weight z-scores were increasing through childhood (Table 2, Figure 3.). Median weight z-scores, calculated using the Lodz and USCDC2000 weight references lie closer to zero in boys (0.01) and girls (0.01), respectively (Figure 3). Figure 3 shows the median weight z-score calculated using three weight references plotted against age for boys and girls separately.

**Figure 4:**
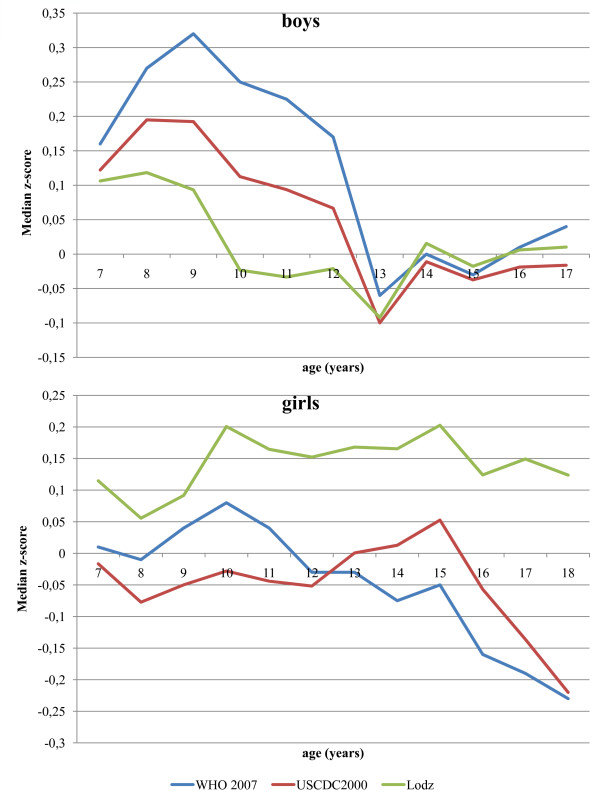
**Polish children (the OLAF study sample) median BMI z-scores calculated using three BMI-for-age references**.

Median BMI z-scores were positive in males in early childhood, decreasing at later ages, and crossing into negativity at the age of 10 and 13 years in the case of z-scores calculated using the Lodz, WHO 2007, and USCDC2000 references, respectively (Figure 4). For all references, the median BMI z-score in boys reached a minimum at age 13 years and then returned to slight positivity, becoming very close to zero (from -0.02 to 0.04 USCDC2000 and WHO 2007, respectively). In the case of girls, the median BMI z-score calculated with the use of WHO 2007 and USCDC2000 was close to zero in early childhood, decreasing in adolescence and reaching minimum values at age 18 years. The median BMI z-score calculated with the use of the Lodz reference fluctuated between 0.05 and 0.2 over the age-range studied (Figure 4).

## Discussion

Periodic health examinations of children and adolescents have been a part of preventive child health programmes in many countries, including Poland. At the examination, it is required to assess centiles of height, weight, and BMI for a child. Screening children's height seems to be cost-effective in detecting height-related disorders and for the detection of other undiagnosed conditions [13]. Growth charts should allow for a precise diagnosis of stunting and disturbances of weight-to-height proportion(s) (underweight and overweight) [14,15]. Currently, the function of growth charts with regard to disturbances of weight is of increasing importance for public health. This is due to the rapidly rising prevalence and magnitude of childhood obesity, which is linked to the incidence of metabolic syndrome [16]. Available growth references have several limitations. In 1995, the WHO Expert Committee established guidelines for preparation of the international growth standards (the sample should comprise healthy children with unconstrained growth, deriving from developed and developing countries; the sample should be characterised by: absence of secular trends, sufficient size, determined cut-off with adequate specificity and sensitivity) [17]. Both the WHO 2007 and USCDC2000 growth references were based mainly on cross-sectional samples with sparse longitudinal data. The influence of a secular trends could not be ruled out.

Our findings demonstrate differences between Polish regional growth charts and the charts based on samples from the USA. The WHO 2007 reference for children aged 5-19 years is based on the 1977 National Center for Health Statistics NCHS/WHO data set, which is used in part in the USCDC2000 reference, but the CDC reference includes some more recent data sets as well. For this reason, the mean z-scores shown in Figure 1 are similar for the WHO 2007 and USCDC2000, with the USCDC2000 score being slightly lower. The comparison shows that mean height z-scores calculated with the WHO 2007 and USCDC2000 height references significantly diverged from zero (means and confidence intervals), whereas mean height z-scores calculated using local Polish growth references, matched zero better. Clinically and statistically important differences between the height of the sampled Polish children and of the US reference population should impose caution when interpreting Polish children's blood pressure using the Fourth Task Force report tables [18] constructed for age and height centiles. Estimation of the height centiles of Polish children using Polish growth references will provide substantially different results than if the WHO 2007 or USCDC2000 growth references are used. As a consequence of this, the cut-off for high blood pressure would differ for a given height.

The observed differences may be attributed to both socioeconomic and genetic factors [19,20]. The analysis performed by Haas et al. clearly showed that pre-pubertal children of most populations grew similarly, but significant differences were revealed in the pubertal stages, in which the European population diverged from the WHO standard by revealing higher values [20]. Surprisingly, our results showed a partially opposite tendency, with lower mean z-scores in pubertal (0.3) than in younger male subjects (0.5). The cross-sectional design of the OLAF study and the influence of improving economic status in Poland (over the last few years) could be an explanation for this observation.

Experts agree that children and adolescents grow similarly when exposed to similar external conditions of growth. The influence of genetic factors might be postulated to some degree as well [17].

Secular trends, body composition, and sexual maturation are critical determinants in the interpretation of anthropometric measures. These constitute the main obstacles in the development of both local and universal growth charts. The OLAF study was designed closer to WHO guidelines to be more applicable for the Polish population. Some of the shortcomings mentioned above cannot be ruled out, however. The data from the Czech Republic reported by Vignerova showed that country-wide growth charts should be updated every 10 years with no significant changes in Body Mass Index or weight-for-height normal values [21]. This strategy may prevent inadequate assessment of overweight and obesity. Moreover, the authors reported that the secular trend in the Czech Republic stopped (except for adolescents). Presumably, this observation might be extrapolated to Poland (a neighbouring country). These findings are not surprising, but of essential clinical relevance. Application of universal growth references or the growth reference of another country to a specific population can lead to underestimation or overestimation of the real rate of growth retardation [22,23]. Additionally, it may negatively influence early detection of other measures related to height-for-age [24].

It is noteworthy that the use of local references constructed with mean and SD only, without accounting for weight and BMI skewness, will give misleading results. For example, the calculation of BMI z-scores using the Warszawa (the most popular in Poland), Poznan and Krakow references resulted in several values greater than six (data not presented in this paper), which are considered implausible by others [25].

In girls from the contemporary sample of Polish children and adolescents, median BMI z-scores calculated using international BMI-for-age references, were closer to zero than with the Lodz standard. In boys, all three compared BMI standards revealed an interesting pattern of median BMI z-score changes from childhood into adolescence. In the age range from 7 to 9 years, median z-scores were positive (more so in the case of the international reference compared with the Polish reference), decreasing into adolescence (age from 13 to 18 years). Much the same pattern of BMI change from childhood to adolescence has recently been reported for three historical British national birth cohorts [26]. The contemporary sample of Polish boys differs in terms of BMI distribution from the Lodz growth reference in the age group 7-9 years and from the WHO 2007 and USCDC2000 in the age group 7-12 years. The difference may be explained by an earlier BMI rebound in the reference data and by increasing obesity among young children in Poland. Similar findings were reported for Chinese boys [27].

## Conclusions

In this contemporary sample of Polish school-aged children and adolescents, distributions of height, weight and BMI differed from those of children in the international growth references. These differences should be taken into consideration when the references are applied. The analysis of height, weight, and BMI z-scores using Polish regional references has certain limitations. Determining the validity and applicability of an existing growth reference and developing a reference based on a national representative sample are important public health functions.

## Abbreviations

BMI: body mass index; BCPE: The Box-Cox power exponential; CDC: Centers for Disease Control and Prevention; CI: confidence interval; IQR: inter-quartile range; max.: maximum; min.: minimum; NCHS: National Center for Health Statistics; SD: standard deviation; WHO: World Health Organization;

## Competing interests

The authors declare that they have no competing interests.

## Authors' contributions

ZK conceived the study, conducted field examinations, did statistical analyses, and drafted the manuscript. ML conceived the study and drafted the manuscript. MT conducted field examinations and drafted the manuscript. AR conceived the study, conducted field examinations and drafted the manuscript. KB conceived the study, conducted field examinations and did statistical analyses. AG conducted field examinations, did statistical analyses. AS conducted field examinations and drafted the manuscript. BG collected, assembled and processed data, and did statistical analyses. EN collected, assembled and processed data. HP conceived the study, did statistical analyses and drafted the manuscript. All authors contributed to the drafting and revisions of the manuscript. All authors read and approved the revised manuscript.

## Pre-publication history

The pre-publication history for this paper can be accessed here:

http://www.biomedcentral.com/1471-2458/10/109/prepub

## Supplementary Material

Additional file 1 **Table S1 - Polish children and adolescents (the OLAF study sample) height, weight, and BMI by sex and age**. table provides descriptive statistics of height (mean, SD, min., max.), weight and BMI (median, IQR, min., max.) by sex and age. Click here for file

Additional file 2 **Table S2 - Polish children and adolescents (the OLAF study sample) 95% CIs of height mean z-scores by age according to the growth chart**. data provided represent 95% confidence intervals of boys' and girls' height mean z-scores according to the six compared growth charts. Click here for file
