# Supplementary material for: The height-, weight-, and BMI-for-age of Polish school-aged children and adolescents relative to international and local growth references
Source: BMC Public Health. 2010 Mar 4;10:109. doi: 10.1186/1471-2458-10-109 (PMC2837854; doi:10.1186/1471-2458-10-109)
Supplement: Additional file 1 — Table S1 - Polish children and adolescents (the OLAF study sample) height, weight, and BMI by sex and age. table provides descriptive statistics of height (mean, SD, min., max.), weight and BMI (median, IQR, min., max.) by sex and age. [file 1471-2458-10-109-S1.DOC]

Table S1. Polish children and adolescents (the OLAF study sample) height, weight, and BMI by sex and age

| sex | age (years) | height (cm) | | | | |  | weight (kg) | | | | |  | BMI (kg/m^2^) | | | |
| --- | --- | --- | --- | --- | --- | --- | --- | --- | --- | --- | --- | --- | --- | --- | --- | --- | --- |
|  |  | N | Min. | Mean | Max. | SD |  | N | Min. | Median | Max. | IQR |  | Min. | Median | Max. | IQR |
| boys | 7 | 380 | 109.8 | 124.9 | 140.1 | 5.4 |  | 380 | 16.0 | 24.3 | 50.4 | 5.8 |  | 12.2 | 15.6 | 28.1 | 2.5 |
|  | 8 | 557 | 110.5 | 130.6 | 151.7 | 5.7 |  | 557 | 19.2 | 27.4 | 59.5 | 6.8 |  | 12.8 | 16.0 | 27.4 | 3.0 |
|  | 9 | 583 | 117.4 | 136.3 | 157.0 | 6.2 |  | 583 | 18.4 | 30.7 | 68.8 | 9.0 |  | 12.3 | 16.5 | 31.2 | 3.4 |
|  | 10 | 516 | 125.3 | 141.5 | 160.2 | 6.8 |  | 516 | 20.6 | 34.3 | 69.3 | 11.1 |  | 11.9 | 17.1 | 29.7 | 3.9 |
|  | 11 | 526 | 130.4 | 146.7 | 171.5 | 6.3 |  | 526 | 24.0 | 38.0 | 88.5 | 10.4 |  | 12.9 | 17.4 | 38.2 | 3.9 |
|  | 12 | 522 | 130.2 | 152.6 | 176.4 | 7.7 |  | 522 | 24.5 | 42.2 | 87.5 | 14.4 |  | 12.5 | 18.0 | 31.5 | 4.5 |
|  | 13 | 513 | 137.2 | 160.3 | 187.2 | 8.8 |  | 513 | 26.5 | 48.0 | 98.5 | 15.5 |  | 12.7 | 18.6 | 32.2 | 4.3 |
|  | 14 | 523 | 139.2 | 166.8 | 186.4 | 8.5 |  | 523 | 31.3 | 53.8 | 128.0 | 14.4 |  | 13.7 | 19.0 | 40.7 | 3.6 |
|  | 15 | 573 | 148.6 | 172.8 | 194.5 | 7.5 |  | 573 | 36.4 | 60.1 | 127.3 | 14.3 |  | 14.3 | 19.9 | 45.1 | 3.4 |
|  | 16 | 498 | 153.7 | 175.5 | 197.8 | 6.8 |  | 497 | 39.3 | 63.1 | 117.7 | 13.2 |  | 13.5 | 20.4 | 35.5 | 3.5 |
|  | 17 | 518 | 155.1 | 178.1 | 196.4 | 6.6 |  | 518 | 45.5 | 67.2 | 126.2 | 13.6 |  | 15.2 | 21.1 | 38.8 | 3.9 |
|  | 18 | 518 | 156.0 | 178.5 | 197.3 | 6.6 |  | 518 | 45.4 | 70.0 | 118.2 | 14.7 |  | 16.2 | 21.7 | 36.3 | 4.1 |
|  |  |  |  |  |  |  |  |  |  |  |  |  |  |  |  |  |  |
| girls | 7 | 317 | 106.7 | 123.6 | 140.2 | 5.6 |  | 317 | 14.3 | 24.0 | 45.3 | 6.1 |  | 11.6 | 15.5 | 25.8 | 2.7 |
|  | 8 | 575 | 114.0 | 129.1 | 149.5 | 5.9 |  | 575 | 16.1 | 26.1 | 52.6 | 6.8 |  | 11.4 | 15.6 | 26.0 | 2.8 |
|  | 9 | 551 | 116.5 | 135.1 | 157.6 | 6.3 |  | 551 | 19.4 | 29.4 | 69.7 | 8.7 |  | 11.7 | 16.1 | 31.0 | 3.5 |
|  | 10 | 583 | 120.0 | 140.7 | 168.2 | 6.9 |  | 583 | 20.0 | 33.3 | 73.8 | 10.1 |  | 12.4 | 16.8 | 29.2 | 3.7 |
|  | 11 | 535 | 123.2 | 146.8 | 171.4 | 7.9 |  | 535 | 20.8 | 37.7 | 77.1 | 12.4 |  | 12.1 | 17.3 | 32.4 | 3.7 |
|  | 12 | 499 | 133.7 | 153.4 | 174.8 | 7.1 |  | 499 | 23.8 | 42.6 | 96.4 | 12.2 |  | 12.7 | 17.9 | 37.0 | 3.8 |
|  | 13 | 503 | 137.1 | 159.1 | 179.4 | 6.3 |  | 502 | 27.5 | 47.8 | 97.0 | 12.1 |  | 12.9 | 18.7 | 38.7 | 3.8 |
|  | 14 | 598 | 145.0 | 162.4 | 180.6 | 6.2 |  | 598 | 26.7 | 51.7 | 116.2 | 11.3 |  | 12.7 | 19.4 | 46.3 | 3.8 |
|  | 15 | 530 | 144.2 | 163.6 | 183.3 | 6.1 |  | 530 | 36.2 | 53.3 | 98.4 | 10.9 |  | 14.9 | 20.1 | 34.6 | 3.3 |
|  | 16 | 582 | 145.5 | 164.5 | 182.6 | 6.0 |  | 581 | 36.7 | 54.7 | 95.9 | 11.4 |  | 13.9 | 20.2 | 34.0 | 3.6 |
|  | 17 | 642 | 145.1 | 164.7 | 181.8 | 5.7 |  | 642 | 39.2 | 55.8 | 108.8 | 10.6 |  | 14.8 | 20.5 | 35.4 | 3.3 |
|  | 18 | 643 | 145.3 | 165.1 | 182.9 | 6.1 |  | 642 | 41.1 | 56.1 | 119.6 | 10.2 |  | 15.7 | 20.6 | 45.3 | 3.1 |
