# Supplementary material for: The height-, weight-, and BMI-for-age of Polish school-aged children and adolescents relative to international and local growth references
Source: BMC Public Health. 2010 Mar 4;10:109. doi: 10.1186/1471-2458-10-109 (PMC2837854; doi:10.1186/1471-2458-10-109)
Supplement: Additional file 2 — Table S2 - Polish children and adolescents (the OLAF study sample) 95% CIs of height mean z-scores by age according to the growth chart. data provided represent 95% confidence intervals of boys' and girls' height mean z-scores according to the six compared growth charts. [file 1471-2458-10-109-S2.DOC]

Table S2 - Polish children and adolescents (the OLAF study sample) 95% CIs of height mean z-scores by age according to the growth chart

| **sex/age** | **N** | **WHO 2007** | | **USCDC2000** | | **Krakow** | | **Warszawa** | | **Poznan** | | **Lodz** | |
| --- | --- | --- | --- | --- | --- | --- | --- | --- | --- | --- | --- | --- | --- |
| **boys** |  | lower CI | upper CI | lower CI | upper CI | lower CI | upper CI | lower CI | upper CI | lower CI | upper CI | lower CI | upper CI |
| **7** | 380 | 0.43 | 0.63 | 0.45 | 0.65 | -0.06 | 0.17 | 0.04 | 0.25 | -0.23 | -0.02 | 0.13 | 0.33 |
| **8** | 557 | 0.50 | 0.66 | 0.41 | 0.57 | 0.11 | 0.27 | 0.17 | 0.33 | -0.09 | 0.09 | 0.16 | 0.33 |
| **9** | 583 | 0.53 | 0.69 | 0.39 | 0.55 | 0.29 | 0.46 | -0.02 | 0.17 | -0.03 | 0.16 | 0.09 | 0.25 |
| **10** | 516 | 0.51 | 0.69 | 0.38 | 0.56 | 0.00 | 0.19 | -0.04 | 0.14 | 0.02 | 0.22 | 0.01 | 0.19 |
| **11** | 526 | 0.49 | 0.64 | 0.43 | 0.57 | 0.05 | 0.20 | 0.10 | 0.27 | -0.06 | 0.11 | 0.04 | 0.20 |
| **12** | 522 | 0.40 | 0.58 | 0.40 | 0.57 | 0.15 | 0.33 | -0.08 | 0.13 | -0.04 | 0.15 | 0.05 | 0.23 |
| **13** | 513 | 0.47 | 0.66 | 0.47 | 0.65 | 0.14 | 0.31 | -0.01 | 0.17 | 0.09 | 0.29 | 0.15 | 0.36 |
| **14** | 523 | 0.38 | 0.57 | 0.34 | 0.53 | 0.04 | 0.22 | 0.03 | 0.19 | -0.08 | 0.10 | -0.06 | 0.14 |
| **15** | 573 | 0.42 | 0.57 | 0.34 | 0.50 | 0.05 | 0.21 | 0.09 | 0.27 | 0.10 | 0.27 | -0.07 | 0.10 |
| **16** | 498 | 0.27 | 0.42 | 0.23 | 0.39 | 0.00 | 0.18 | -0.22 | -0.04 | -0.11 | 0.08 | -0.12 | 0.05 |
| **17** | 518 | 0.31 | 0.46 | 0.32 | 0.48 | 0.19 | 0.37 | -0.13 | 0.05 | 0.00 | 0.18 | 0.08 | 0.25 |
| **18** | 518 | 0.25 | 0.40 | 0.27 | 0.42 | -0.01 | 0.16 | -0.08 | 0.10 | -0.12 | 0.06 | 0.11 | 0.29 |
| **girls** | | | | | | | | | | | | | |
| **7** | 317 | 0.29 | 0.51 | 0.18 | 0.39 | 0.14 | 0.36 | -0.06 | 0.16 | -0.28 | -0.04 | -0.04 | 0.19 |
| **8** | 575 | 0.34 | 0.50 | 0.19 | 0.35 | -0.15 | 0.01 | -0.13 | 0.06 | -0.11 | 0.07 | 0.02 | 0.18 |
| **9** | 551 | 0.36 | 0.52 | 0.30 | 0.46 | 0.13 | 0.32 | -0.09 | 0.09 | -0.08 | 0.12 | 0.11 | 0.28 |
| **10** | 583 | 0.25 | 0.41 | 0.34 | 0.50 | 0.03 | 0.20 | -0.02 | 0.15 | -0.03 | 0.15 | 0.03 | 0.20 |
| **11** | 535 | 0.18 | 0.37 | 0.33 | 0.54 | 0.10 | 0.30 | -0.17 | 0.02 | -0.17 | 0.03 | -0.04 | 0.17 |
| **12** | 499 | 0.24 | 0.41 | 0.27 | 0.44 | 0.00 | 0.18 | 0.00 | 0.19 | -0.03 | 0.16 | 0.03 | 0.22 |
| **13** | 503 | 0.31 | 0.47 | 0.24 | 0.40 | -0.03 | 0.14 | -0.07 | 0.11 | -0.09 | 0.10 | 0.13 | 0.30 |
| **14** | 598 | 0.30 | 0.45 | 0.24 | 0.40 | 0.04 | 0.21 | 0.01 | 0.17 | -0.08 | 0.10 | 0.14 | 0.30 |
| **15** | 530 | 0.22 | 0.37 | 0.20 | 0.36 | -0.15 | 0.02 | -0.03 | 0.15 | -0.19 | 0.01 | 0.06 | 0.23 |
| **16** | 582 | 0.22 | 0.36 | 0.23 | 0.38 | -0.11 | 0.07 | -0.09 | 0.08 | -0.15 | 0.03 | 0.07 | 0.23 |
| **17** | 642 | 0.21 | 0.34 | 0.21 | 0.34 | 0.02 | 0.17 | -0.17 | 0.00 | -0.31 | -0.14 | 0.07 | 0.22 |
| **18** | 643 | 0.25 | 0.39 | 0.24 | 0.39 | 0.08 | 0.23 | -0.13 | 0.03 | -0.31 | -0.14 | 0.16 | 0.32 |
